# Substance use disorders, severe mental illness and risk of reoffending among women released from prison: a national cohort study

**DOI:** 10.1038/s41598-025-00215-y

**Published:** 2025-05-03

**Authors:** Vegard Svendsen, Marianne Riksheim Stavseth, Torbjørn Skardhamar, Ingrid Amalia Havnes, Anne Bukten

**Affiliations:** 1 Norwegian Centre for Addiction Research, University of Oslo, Oslo, Norway; 2 Division of Mental Health and Addiction, Oslo University Hospital, Oslo, Norway; 3 Department of Sociology and Human Geography, University of Oslo, Oslo, Norway; 4 Institute of Clinical Medicine, Faculty of Medicine, University of Oslo, Oslo, Norway

**Keywords:** Substance use disorders, Recidivism, Dual disorders, Psychiatric comorbidity, Prison, Women’s health, Epidemiology, Psychiatric disorders

## Abstract

Women with a clinical history of a severe mental illness (SMI) or multiple mental disorders involving substance use disorder (SUD) might be at increased risk of re-offending after release from prison. This retrospective cohort study merged data from the Norwegian prison release study (nPRIS) with other registry sources. All women released from a Norwegian prison between 2011–2019 were followed for two years after date of release. Adjusting for several known risk factors, we used logistic regression to estimate risk of violent (VR), drug-related (DR) or other reoffending (ORE) after release among women with a history of SUD and severe mental illness (SUD-SMI), SMI, SUD and other mental illness (SUD-OMI) or a history of multiple SUDs (POLY-SUD) in comparison to a reference population with no history of such disorders or combination of disorders. Relative to the reference population, after adjusting for other relevant risk factors, women with a history of SUD-SMI (OR_VR_ 2.27, 95% CI 1.37–3.76; OR_DR_ 2.10, 95% CI 1.49–2.95), SUD-OMI (OR_VR_ 1.81, 95% CI 1.11–2.47; OR_DR_ 1.53, 95% CI 1.11–2.10) or POLY-SUD (OR_VR_ 1.87, 95% CI 1.04–3.31; OR_DR_ 2.51, 95% CI 1.80–3.49) were at significantly greater risk of violent and drug-related reoffending. Women with a history of SMI were at significantly greater risk of other reoffending (OR_ORE_ 2.64, 95% CI 1.21–5.30). Women with a clinical history of a severe mental illness (SMI) or multiple mental disorders involving substance use disorder (SUD) have an elevated risk reoffending.

## Introduction

Recidivism, commonly defined as the tendency of a convicted person to reoffend, is a key metric of any correctional system that aims to rehabilitate and reintegrate criminal offenders^1^. It is also a useful proxy for a variety of negative health and social outcomes^2,3^ and occurs at a high cost to society^4,5^. While recidivism is frequently researched in the correctional literature, there is a dearth of gender specific and gender informed investigations^6–8^.

Persons with a history of severe mental illness (SMI), multiple substance use disorders (POLY-SUD) or some combination of both substance use disorders (SUD) and mental illness (MI) are at increased risk of poor physical and mental health, reduced functioning and short life expectancy^9–15^. These are disorders or combinations of disorders which are associated with complex and unmet treatment needs^16–18^, victimization, stigma and social drift^19,20^.

Histories of SMI, POLY-SUD or SUD-MI are also associated with an increased risk of committing violent crime^21–24^ and repeat offending^25–30^ and are overrepresented among people in prison^31,32^. It has also been emphasized that personality disorders (PD), and particularly borderline (BPD) and antisocial PD (APD), which are also highly prevalent in incarcerated populations^33^, play a central role in understanding psychiatric comorbidities and reoffending risk in this context^34,35^.

Relatedly, a history of substance psychosis (SIP) is also strongly associated with (violent) re-offending^36^, and particularly in women^37^. There is growing evidence for high transition rates from SIP to an SMI diagnosis^38,39^ suggesting that SIP might represent an important proxy for potentially undiagnosed SMI.

While more men than women tend to use illicit drugs and alcohol, the progression of SUD and treatment seeking tend to be more accelerated in women and more likely to also involve psychiatric comorbidity^40,41^. Women who experience incarceration often present with high rates of substance use problems and mental health needs, with psychiatric disorders and comorbidities being both highly and more prevalent than among men^42–48^. In Norway, the majority of women entering prison have a recent history of at least one psychiatric diagnosis, including SUDs, and 38%, versus 24% of men, have a lifetime history of SUD and other mental illness (MI)^45^.

The disproportional burden of mental illness among women who experience incarceration could indicate that psychiatric disorders have particular relevance for women’s risk of involvement with the criminal justice system^37,49^ and especially for violent crime^34,50–53^.

A recent meta-analysis found that individual disorders such as depression and post-traumatic stress disorder (PTSD) were significantly associated with women’s risk of reoffending^49^. However, there is the lack of gender-specific investigations into the risk relevance of higher order combinations of psychiatric disorders, especially in the European context. Given the high rates of comorbidities among women who experience incarceration, this constitutes a major limitation of the current literature.

Despite growing attention to the role of psychiatric comorbidities in the forensic literature, there is a shortage of studies addressing the risk-relevance of SUD and MI among women. To our knowledge, the recent papers from Mayer et al.^51^ and Wolf et al.^53^, both from a German forensic treatment context, are the only studies to investigate specifically the association between a history of SUD and MI and later re-offending among women. Both studies found a significant effect of comorbid SUD and personality disorder on violent re-offending, though with limited adjustment for other risk factors.

### Context

Scandinavian countries have some of the smallest per capita prison populations in the world^54^. These are in turn highly selected and at-risk populations^55^, largely and increasingly characterised by deprivation and marginalization^56,57^. In Norway, especially over the last decade and a half, several societal trends and changes in correctional practice have co-occurred, such as a general reduction in national crime rates, downsizing of inpatient public mental health services and an increasing use of alternative sanctions to incarceration, in particular electronic monitoring. As a result, the relatively small group that still ends up in prison tends to comprise individuals who have either committed more severe offences or, for other reasons, are not suitable candidates for alternative sanctions^58^.

One unintended, yet evident consequence of these developments over time, especially among women, is the increasing prevalence of psychiatric disorders among new entries to prison^45^. Yet, little is known about the long-term effects or practical implications of this reality. Women involved with the justice system represent a small, vulnerable and under-researched minority. There is a pressing need for more knowledge about how mental illness contributes to criminogenic risk and treatment needs among women in the Scandinavian context.

### Aims

With access to high quality national registry data, we suggest a retrospective cohort-study to investigate the association between SMI or a history of multiple mental disorders and re-offending in all women released from Norwegian prisons over a nine-year period. Specifically, we wanted to test the association between having a clinical history of either SMI or multiple mental disorders involving SUDs upon entry to prison and committing a new criminal offence resulting in a conditional or unconditional prison term within two years after release, while adjusting for risk factors identified in previous literature. By stratifying our analysis by type of criminal offending, we also wanted to estimate whether various mental health profiles are differently associated with violent, drug related and other re-offending in this population.

## Methods

### Data sources

This study is part of the PriSUD-project^59^ and utilizes data from the Norwegian Prison Release Study (nPRIS)^60^, which holds demographic and correctional data on all persons released from Norwegian prisons between Jan 1st 2000–Dec 31st 2022. For persons within this cohort also holding a Norwegian personal identification number (PIN), individual level data coupling with other national registries is possible. Since 2008, the Norwegian Patient Registry (NPR) has collected data from all contacts with the specialized health care services by adults aged 18 or older—but only with complete data (i.e. covering all service areas) since 2009^61^. From the NPR we used date of contact, as well as primary and secondary diagnosis recorded according to the *International statistical classification of diseases and related health problems, tenth revision (ICD-10)*^62^. From Statistics Norway (SSB; a national institute responsible for collecting and producing various statistics related to economy and demographics on all citizens), we used data related to motherhood status, as well as on social benefits, education and criminal offending. We collected date of death data from the Norwegian cause of death registry (NCD).

### Sample

The population of interest was women who had experienced incarceration and had a clinical history of MI upon entry to prison in Norway. Given the PIN requisite and the time and age limitations on the availability of health data, and since we wanted a minimum two-year window of complete NPR coverage for all women prior to incarceration as well as a uniform two-year follow up after release, the study population was defined as all women in the nPRIS cohort with a valid PIN who, at a minimum age of 20, experienced at least one day of incarceration (at any security level or on remand) between January 1, 2011 and December 30, 2019, and who were released no later than December 31, 2019. The level of analysis was the index (i.e. first recorded) incarceration within the study period.

### Measures

#### Grouping variables

Diagnoses in Chapter V in the ICD-10 were used to identify women in the study population with any history (i.e., between 2008 and entry to index incarceration) of MI diagnosed in the specialist health service. These diagnoses included substance use disorders (F10-19), psychotic disorders (F20-29), mood disorders (F30-39), anxiety disorders (F40-49), behavioral syndromes associated with physical factors (F50-59), personality disorders (PD) (F60-69), intellectual and developmental disorders (F70-89), behavioural disorders with early onset (F90-98) or unspecified mental disorders (F99). For all clinical data, we included both primary and secondary diagnosis, with the only temporal requirement that any diagnosis was recorded prior to entry to index incarceration.

Types of mental illness histories/profiles were defined in four mutually exclusive categories based on ICD-10 codes:

1. **SUD-SMI:** any SUD [F10-19, excl. F17] and any SMI (psychosis, bipolar disorder or major depression; [F20-31, F32.2, F32.3, F33.2, F33.3] or any history of substance induced psychosis (SIP) [F1X.5]) or borderline (BPD) or antisocial PD (APD) [F60.2, F60.3])
2. **SMI:** any SMI but no history of SUD
3. **SUD-OMI:** any SUD and any other mental illness (OMI) [F32-F99, excl. F32.2, F32.3, F33.2, F33.3, F60.2, F60.3]) but no history of SIP.
4. **POLY-SUD:** any history of more than one SUD [F10-18, excl. F17] or any F19 diagnosis but no history of any SMI or OMI

Women qualifying for either SUD-SMI, SMI, SUD-OMI or POLY-SUD were compared with a reference category, which comprised all other women in the study population and included women with either no history of any psychiatric disorder, only one psychiatric disorder (including SUD [F10-18]) or any combination of two or more diagnoses but no history of SUD-SMI, SMI, SUD-OMI or POLY-SUD. In accordance with previous research^33^ BPD and APD were conceptualized as SMIs. As was any history of SIP.

#### Covariates

Selection of covariates was guided by risk factors identified in previous research. Their inclusion and operationalisation were also dependent on availability in the registries and subject to varying coverage (see *Data Sources* above and Table 1). Socioeconomic covariates included criminal history (i.e., number of violent,   drug-related or other offences to result in a conditional or unconditional prison sentence prior to index incarceration), prison security level at release (high or low), release on probation (yes or no), civil status and whether partner or parent have any history of incarceration^25,63^; history of receiving social welfare or benefits^64^; level of education^65^; motherhood (i.e., having own minor children and whether one is cohabiting with these)^66,67^ (Table 1).

**Table 1:** Description of socioeconomic variables and time frame for observation.

Variable	Description/definition	Time frame
Criminal history		*Between January 1, 2000 and date of index incarceration*
Number of prior offences	Number of recorded prior violent, drug-related or other offences resulting in a conditional or unconditional prison sentence	
Civil status^1^		*In the calendar year prior to index incarceration*
No partner^2^	Not married or cohabiting	
Partner	Married or cohabiting	
Partner with history of incarceration	Married or cohabiting with partner with history of at least one incarceration (between January 1, 2000 and index incarceration)	
Parent with incarceration history^1^	Mother or father with or without history of at least one incarceration (between January 1, 2000 and index incarceration)	*In the calendar year prior to index incarceration*
Age	Age in years, operationalized in the analysis as five-year age groups	*At entry to index incarceration*
Motherhood^1^		*In the calendar year prior to index incarceration*
No own minor children^2^	No current record of any own minor children (< 16 years of age)	
Have and live with any own minor children	Number of recorded minor children is equal to or smaller than the number of children below age 18 in the household	
Have but do not live with all own minor children	Number of recorded own minor children is higher than the number of children below age 18 in the household	
Education level^1^	Highest recorded level of completed education either i) above or ii) equal to or below year 10. Any record of completed education, but with level unknown was recorded as being below year 10	*In the calendar year prior to index incarceration*
Recipient of social welfare or benefits^1^		*In the calendar year prior to index incarceration*
No^2^	No record or no amount of received social welfare	
Yes, low	Received social welfare, but below median of all recipients	
Yes, high	Received social welfare, above median of all recipients	

^1^Levels are mutually exclusive.

^2^Reference level in regression analysis.

We also included other potentially risk-relevant clinical characteristics not captured by the grouping variables. This included any history of attention-deficit hyperactivity disorder (ADHD) [F90]^68^, post-traumatic stress disorder (PTSD) [F43]^49^, self-harm^64,69^ or head trauma^29,70,71^. The latter two were defined as any record of hospitalization for self-harm [X60-79, 81–84, Y10-39, Y10-34, Y87] or traumatic brain injury (TBI) [S00-9]). For descriptive purposes, we also report the prevalence of psychiatric disorders according to the main chapters of the ICD-10 Chapter V, as well as SIP prevalence.

#### Outcomes

The primary outcome, reoffending within two years after release which resulted in a new conditional or unconditional prison term, was classified hierarchically and based on type of reoffending as defined according to SSBs crime index^72^. Women who committed a violent reoffence were categorized accordingly, regardless of whether they at any time during follow up also committed a drug related or other type of reoffence. Women who committed a drug related reoffence, but no violent reoffence, was categorized accordingly, regardless of any non-drug or non-violent type of reoffence. Women who committed a reoffence that was not violent, nor drug related, and also had no record of a violent or drug related reoffence, were categorized as committing another type or reoffence. The rationale for this hierarchical approach was to adjust for the potential high degree of overlap between types of reoffending and to avoid inflated effect sizes due to repeated measurements particularly among certain high-risk individuals presenting with multiple type of reoffences.

#### Statistical analysis

All women were followed for up to two years after date of release from index incarceration. For each diagnostic category, we report number of individuals who died during follow-up. Stratified by type of re-offence (violent, drug related or other)), risk of reoffending was estimated using unadjusted and adjusted odds ratios (ORs) using logistic regression.

Missing values were handled by complete case analysis. The only covariates with missing values were education level (n = 58), partner (n = 2), prison security level at release (n = 1) and parent status (n = 1). A total of 60 observations were excluded from the regression analysis due to missingness.

## Results

A total of 4,573 women with a Norwegian PIN aged 20 or older entered and were released from a Norwegian prison between 2011 and 2019 (Table 2). They were typically around 37 years of age upon entry to prison and experienced incarcerations that were most commonly shorter than one month in length. In the year prior to incarceration, most lived in single person households, either not having or not living with their own minor children. The majority had some education above year 10, but still received some form of social welfare or benefits (Table 2).

**Table 2:** Demographic and socioeconomic characteristics and outcomes for overall cohort (n = 4573) and diagnostic category.

	Overall, n = 4573^1^	SUD-SMI, n = 665^1^	SMI, n = 199^1^	SUD-OMI, n = 927^1^	POLY-SUD, n = 504^1^	Reference, n = 2278^1^
Age	37 (28, 46)	35 (27, 43)	39 (30, 47)	34 (26, 43)	36 (27, 43)	39 (29, 48)
Sentence length	28 (18, 60)	29 (18, 60)	27 (18, 60)	30 (21, 59)	32 (21, 64)	28 (18, 60)
High security release	1823 (40)	347 (52)	93 (47)	370 (40)	214 (42)	799 (35)
Release on probation	235 (5)	39 (6)	9 (5)	55 (6)	31 (6)	101 (4)
Any previous violent offence	1,278 (28)	248 (37)	58 (29)	251 (27)	117 (23)	604 (27)
Any previous drug related offence	2613 (57)	490 (74)	73 (37)	741 (80)	408 (81)	901 (40)
Any previous other offence	2,593 (57)	433 (65)	95 (48)	616 (66)	355 (70)	1,094 (48)
Civil status						
No partner	2353 (51)	403 (61)	94 (47)	545 (59)	316 (63)	995 (44)
Partner	1,553 (34)	167 (25)	86 (43)	216 (23)	83 (16)	1,001 (44)
Partner with incarceration history	665 (15)	95 (14)	19 (10)	166 (18)	105 (21)	280 (12)
Parent with incarceration history	422 (9)	72 (11)	13 (7)	124 (13)	68 (13)	145 (6)
Motherhood						
Have and live with own minor children	793 (17)	65 (10)	41 (21)	102 (11)	28 (6)	557 (24)
Have but do not live with own minor children	1,050 (23)	188 (28)	45 (23)	286 (31)	144 (29)	387 (17)
No children	2730 (60)	412 (62)	113 (57)	539 (58)	332 (66)	1,334 (59)
Any social welfare or benefits	2329 (51)	435 (65)	72 (36)	631 (68)	396 (79)	795 (35)
Low education	1269 (28)	190 (29)	47 (24)	280 (30)	174 (35)	578 (26)
Died during follow-up	57 (1)	11 (2)	5 (3)	16 (2)	9 (2)	16 (1)
Violent reoffence	175 (4)	57 (9)	4 (2)	47 (5)	24 (5)	43 (2)
Drug related reoffence	423 (9)	86 (13)	6 (3)	113 (12)	104 (21)	114 (5)
Other reoffence	116 (3)	20 (3)	10 (5)	28 (3)	16 (3)	42 (2)

^1^All values are median (IQR) or n (%).

*SUD* substance use disorder, *MI* mental illness, *SMI* severe mental illness.

Of the total study population, about 50% (n = 2,295) had a history of SMI or multiple disorders involving SUD or SUDs. Of these, 15% (n = 665) had SUD and a history of SMI. Four percent (n = 199) had SMI but no history of SUD. The majority (n = 927, 20%) involved SUD and mental illnesses other than conditions qualifying for SMI (OMI). Eleven percent (n = 504) had multiple SUDs (POLY-SUD) but no history of SMI or OMI (Table 3).

**Table 3:** Clinical characteristics for overall cohort (n = 4573) and by diagnostic category.

	Overall, n = 4573^1^	SUD-SMI, n = 665^1^	SMI, n = 199^1^	SUD-OMI, n = 927^1^	POLY-SUD, n = 504^1^	Reference, n = 2278^1^
Any SUD (F10-19)	2357 (52)	665 (100)	–	927 (100)	504 (100)	261 (11)
DUD	2021 (44)	595 (89)	–	793 (86)	504 (100)	129 (6)
AUD	953 (21)	327 (49)	–	380 (41)	114 (23)	132 (6)
Any SIP	229 (5)	229 (34)	–	–	–	–
Psychotic disorders (F20-29)	205 (4)	165 (25)	40 (20)	–	–	–
Schizophrenia (F20)	67 (1)	55 (8)	12 (6)	–	–	–
Mood disorders (F30-39)	1125 (25)	394 (59)	147 (74)	342 (37)	–	242 (11)
Bipolar	207 (5)	156 (23)	51 (26)	–	–	–
Depression	1015 (22)	314 (47)	117 (59)	342 (37)	–	242 (11)
Anxiety disorders (F40-48)	1365 (30)	377 (57)	114 (57)	545 (59)	–	329 (14)
PTSD	364 (8)	130 (20)	33 (17)	129 (14)	–	72 (3)
Behavioral syndromes associated with physical factors (F50-59)	155 (3)	58 (9)	11 (6)	62 (7)	–	24 (1)
Personality disorders (F60-69)	486 (11)	315 (47)	78 (39)	65 (7)	–	28 (1)
Borderline	331 (7)	266 (40)	65 (33)	–	–	–
Antisocial	36 (1)	32 (5)	4 (2)	–	–	–
Intellectual and developmental disorders (F70-89)	74 (2)	21 (3)	11 (6)	24 (3)	-	18 (1)
Behavioral disorders with early onset (F90-98)	515 (11)	157 (24)	24 (12)	249 (27)	–	85 (4)
ADHD	478 (10)	146 (22)	21 (11)	232 (25)	–	79 (3)
Unspecified mental disorder (F99)	452 (10)	135 (20)	32 (16)	204 (22)	–	81 (4)
Any hospitalization for self-harm	327 (7)	145 (22)	20 (10)	96 (10)	34 (7)	32 (1)
Any hospitalization for TBI	427 (9)	134 (20)	11 (6)	120 (13)	53 (11)	109 (5)

*SUD* substance use disorder, *MI* mental illness, *DUD* drug use disorder, *AUD* alcohol use disorder, *SIP* substance induced psychosis, *PTSD* post-traumatic stress disorder, *ADHD* attention-deficit hyperactivity disorder, *TBI* traumatic brain injury.

^1^All values are n (%).

Within two years following release from index incarceration, four, nine and three percent of the total study population had committed a violent (VR), drug related (DR) or other type (ORE) of reoffence, respectively.

VR was most common among SUD-SMI (nine percent), DR among POLY-SUD (21%) and ORE among SMI (five percent). While women with SUD-SMI or POLY-SUD constituted 25% of the total study population, they accounted for 46% of VR and 45% of DR (Table 2).

Unadjusted analyses suggest that all diagnostic categories except SMI were associated with increased risk of both VR (Table 4), DR (Table 5) and ORE (Table 6), relative to a reference population of women with no history of SMI or multiple disorders involving SUD or multiple SUDs. SMI was only significantly associated with ORE (Table 6) The overall highest risk was observed for VR and among women with a history of SUD-SMI (Table 4).

**Table 4:** Unadjusted and adjusted logistic regression odds ratios estimating risk of violent reoffending (VR) after release from prison among all women (n = 4513) with a history of SUD-SMI, SMI, SUD-OMI or POLY-SUD in comparison to a reference population without such histories.

	Unadjusted analysis			Adjusted analysis^1^		
	OR	95% CI	*p*	OR	95% CI	*p*
SUD-SMI	4.87	3.25, 7.35	< 0.001	2.27	1.37, 3.76	0.002
SMI	1.07	0.32, 2.67	> 0.9	0.96	0.28, 2,47	> 0.9
SUD-OMI	2.78	1.82, 4.24	< 0.001	1.81	1.11, 2.96	0.022
POLY-SUD	2.60	1.54, 4.29	< 0.001	1.87	1.04, 3.31	0.034

^1^Adjusted for age; number of previous violent, drug related and other offences; security level; probation; cohabitation with own minor children and any partner; whether partner or any parent have been incarcerated; level of education; received social welfare or benefits; history of PTSD or ADHD; any hospitalization for self-harm or TBI.

**Table 5:** Unadjusted and adjusted logistic regression odds ratios estimating risk of drug related reoffending (DR) after release from prison among women (n = 4434) without any record of violent reoffending with a history of SUD-SMI, SMI, SUD-OMI or POLY-SUD in comparison to a reference population without such histories.

	Unadjusted analysis			Adjusted analysis^1^		
	OR	95% CI	*p*	OR	95% CI	*p*
SUD-SMI	2.94	2.19, 3.95	< 0.001	2.10	1.49, 2.95	< 0.001
SMI	0.59	0.23, 1.25	0.2	0.63	0.24, 1.35	0.3
SUD-OMI	2.70	2.06, 3.55	< 0.001	1.53	1.11, 2.10	0.009
POLY-SUD	5.13	3.84, 6.83	< 0.001	2.51	1.80, 3.49	< 0.001

^1^Adjusted for age; number of previous violent, drug related and other offences; security level; probation; cohabitation with own minor children and any partner; whether partner or any parent have been incarcerated; level of education; received social welfare or benefits; history of PTSD or ADHD; any hospitalization for self-harm or TBI.

**Table 6:** Unadjusted and adjusted logistic regression odds ratios estimating risk of other reoffending (ORE) after release from prison among women (n = 4098) without any record of violent and/or drug related reoffending with a history of SUD-SMI, SMI, SUD-OMI or POLY-SUD in comparison to a reference population without such histories.

	Unadjusted analysis			Adjusted analysis^1^		
	OR	95% CI	*p*	OR	95% CI	*p*
SUD-SMI	1.87	1.07, 3.18	0.023	1.40	0.73, 2.61	0.3
SMI	2.79	1.30, 5.43	0.004	2.64	1.21, 5.30	0.009
SUD-OMI	1.81	1.11, 2.93	0.016	1.42	0.81, 2.48	0.2
POLY-SUD	2.04	1.11, 3.59	0.017	1.60	0.80, 3.03	0.2

^1^Adjusted for age; number of previous violent, drug related and other offences; security level; probation; cohabitation with own minor children and any partner; whether partner or any parent have been incarcerated; level of education; received social welfare or benefits; history of PTSD or ADHD; any hospitalization for self-harm or TBI.

After adjusting for other risk-factors, risk estimates for SUD-SMI remained significant for VR (OR_VR_ 2.27, 95% CI 1.37–3.76, Table 4) and DR (OR_DR_ 2.10, 95% CI 1.49–2.95, Table 5), but not for ORE. For SMI, VR and DR remained unsignificant while risk estimate for ORE remained significant (OR_ORE_ 2.64, 95% CI 1.21–5.30, Table 6). For SUD-OMI, risk estimates remained significant for VR (OR_VR_ 1.81, 95% CI 1.11–2.47, Table 4) and DR (OR_DR_ 1.53, 95% CI 1.11–2.10, Table 5), but not for ORE. For POLY-SUD, risk estimates remained significant for VR (OR_VR_ 1.87, 95% CI 1.04–3.31, Table 4) and DR (OR_DR_ 2.51, 95% CI 1.80–3.49, Table 5), but not for ORE.

## Discussion

In a population of 4,573 women followed for two years after release from prison, 50% had a clinical history involving either severe mental illness, multiple disorders involving substance use disorder or a history of multiple substance use disorders upon entry to prison. Multiple substance use disorders, or the combination of substance use disorder (SUD) and severe (SMI) or other mental illness (OMI) was associated with increased risk for both violent and drug related reoffending, even after adjusting for other relevant risk factors. Women with a history of either multiple substance use disorders (POLY-SUD) or both SUD and SMI (SUD-SMI) emerged as a particularly at-risk group. While women with SUD-SMI or POLY-SUD constituted a relative minority of the total study population, they accounted for almost half of the observed violent and drug related reoffence categories. Conversely, the reoffence category which comprised only non-violent and non-drug-related offences were most common among women with only SMI (i.e. no history of any SUD). After adjusting for other risk-factors, SMI was the only diagnostic category for whom risk-estimates for non-violent and non-drug-related reoffending remained significant.

### Comparison of results and methodology with previous literature

Results generally align with previous research suggesting that individuals with a history of SUD and SMI^21,25–27^, and justice involved women with comorbid SUD and PD^51,53^ might be at particular risk of reoffending. Conversely, women SMI but no SUD had a distinctively different reoffending profile compared to women in the other diagnostic categories, which all involved SUD or SUDs.

SMI was significantly associated with only non-violent and non-drug-related reoffences, a pattern which was the inverse for the other groups. We believe this finding reflect, at least in part, the importance of addressing also other concurrent risk-factors, and especially substance use, and their influence on the association between SMI and various types of risk, including violence. Moreover, the relatively low risk of violent reoffending among women with SMI in the current study is also in line with previous research suggesting that the observed association between SMI and violence is subject to important gender differences, with women presenting with much lower incidence of violent crime compared to their male peers^73^.

However, it should also be noted that our definition of SMI was relatively broad and included disorders (i.e. borderline and antisocial PD) and conditions (i.e. substance induced psychosis) which are typically not included in such classifications. This implies some imprecision with regards to capturing the risk relevance of more specific disorders such as schizophrenia and other psychotic disorders. Such disorders are more commonly associated with violence, also among women when compared to general population controls^24^.

The inclusion of any history of SIP as a qualifier for SMI is novel to this study. For some, SIP might indicate an underlying and potentially undiagnosed SMI^38,39^. However, SIP may also be related to high-risk drug use—which in turn is also associated with reoffending^74^. The extent to which SIP represents a reliable proxy for SMI should be further explored in longitudinal studies. Similarly, the inclusion of POLY-SUD as separate category is also relatively unchartered territory. In our study, POLY-SUD emerged as a particularly at-risk group for both violent and drug-related reoffending. While women presenting with POLY-SUD were defined as not having any history of any diagnosed SMI or OMI, they shared many of the same characteristics and markers of marginalization as the women who did. There might be an element of underdiagnosis at play here, and consequently an increased risk of misclassification (see also limitations, below) for this category.

### Social context and implications

In general, results from the current study give weight to the importance of addressing mental health needs, including SUD treatment, among female criminal offenders. Other risk factors for reoffending, such as low socioeconomic status, frequent previous offenses, or a history of diagnosed head injury, self-harm, ADHD and PTSD, were highly prevalent in this population. Still, statistical models in which these were accounted for found a significant association between a clinical history of SUD and MI or multiple SUDs at entry to prison and later reoffending.

However, this association is likely to also be dependent upon other, unmeasured factors. Some of these might be of a social or political nature and their relative influence difficult to estimate or adjust for in a statistical analysis. Other factors might be related to other clinical aspects or specific interventions that could be the subject for future research. For instance, with reference to the former, Norway is a country whose welfare and political system has increasingly allowed, or failed to redress, the (unintentional) criminalization^75^ of certain mental health problems. Measures such as the reduction in number of psychiatric hospital beds, restrictions on civil commitment criteria and the overreliance on law enforcement for handling offenders with mental illness entails that certain groups or individuals who primarily are in need of psychiatric care instead become involved with the criminal justice system^76^. Given the high rates of mental health and substance use problems among justice-involved women^45^, it follows that many are disproportionally likely to be (re)arrested or (re)imprisoned.

Moreover, in a recent report looking at population level data to investigate a variety of outcomes among young criminal offenders, early criminal justice involvement was found to be especially detrimental for women and girls^77^. Women who experience incarceration might also be at particular risk for premature mortality^78^. Some effect of selection bias, in that certain defining vulnerabilities which in turn are disproportionally characteristic of female offenders, might account for some of these gender differences. However, one could also make the case for a widely unfavorable interaction effect between the same vulnerabilities and many negative aspects of incarceration^79^.

Although the SUD prevalence gap between men and women seem to be narrowing^80,81^ women’s pathway into substance use and drug crime might be qualitatively different from men’s^82,83^. Women seem to develop SUD faster than men, have more functional impairment and co-occuring mental illness^40,84^, and are more likely to engage in drug use as a means of self-medication rather than sensation-seeking and experimentation^85,86^. Women tend to be more susceptible to participate in the drug supply chain as a consequence of oppression and vulnerability^87^. Given the extent of victimization in this population^71,88^, an understanding of substance use as a sign of deprivation rather than of deviant social behavior is potentially crucial. The punitive and security-oriented philosophy inherent to the criminal justice system, which typically follows the latter approach, might not be conducive to the process of rehabilitating highly marginalized offenders suffering from post-traumatic stress and other mental health problems.

Conversely, a period of incarceration also represents a window of opportunity, both for detecting and addressing mental health problems as well as other issues related to marginalization^89^. Re-entry programs also play a crucial role in maintaining treatment and facilitating reintegration to society^90^. Also, within this perspective there is a potential range of factors likely to have some moderating effect on the probability of reoffending which were not covered in this study. These include clinical factors, such as symptom severity, access to treatment or treatment adherence^91^, both before, during and immediately after release, but also other in-prison and post-release characteristics, such as access to, and quality of, relevant training and re-entry programs.

In sum, we strongly encourage the deliberation of these findings and perspectives in future policy developments. Specifically, the focus should be on strengthening public psychiatric services as well as on potential correctional diversion strategies^92^ for offenders presenting with severe or complex mental health problems.

We also emphasize the importance of ensuring sufficient mental health care to all persons in and exiting prison, and that these services should integrate both substance use and other mental health treatment. Evidence to suggest an accumulation of the most marginalized offenders in women’s prisons should also be approached with the utmost sincerity^45^, as should the burden of psychiatric comorbidities and other health concerns in this population^93^. Comorbidities involving SUDs are associated with complex treatment needs, poor prognoses and a high risk for a variety of negative outcomes^9–14,16–18^. Several SUDs have chronic courses and early access to treatment may result in a more positive outcome^94^. Confronted with a population with an increasing prevalence of such disorders, it is paramount that women’s correctional institutions are dimensioned and organized to meet the needs of these women.

Future studies should assess potential differences in relevant outcomes between women released from prison and those serving their sentence under alternative sanctions or diversion programs. Such studies should also focus on the effect of receiving mental health treatment or other social interventions (e.g., individual placement and support (IPS)^95^) provided during or after correctional measures.

### Strengths and limitations

The main strength of this study is the unique quality of the underlying data material ^59^, which gave us access to 13 years of consistent observation and high quality registry data on a complete population of women incarcerated in Norway.

Nevertheless, the use of registry data to diagnostically categorize individuals has several limitations. First, our estimates are based on the history of secondary health care utilization in our population and are prone to underestimate the true prevalence of any disorder and potential misclassification. There is an inherently low negative predictive value to registry data, and the absence of a recorded diagnosis cannot be assumed to equal absence of disorder or symptoms. This limitation also applies to most socioeconomic variables.

Moreover, for this study we did not impose a limit on the observation time prior to incarceration. This is prone to introduce some bias in the analysis by allowing a longer coverage for incarcerations that occurred later in the observation period and for women who were older at entry to prison. However, this limitation must be weighed against the bias of potential case misclassification from applying an arbitrary temporal cut-off for qualifying for what may in many instances represent chronic mental health problems.

Another limitation of this study is that we did not adjust for the full range factors likely to have some moderating effect on the probability of reoffending (as discussed above). Moreover, as we required any diagnosis to be recorded prior to entry to prison, we did not account for the possibility that some women were either first diagnosed or even developed psychiatric disorders during or as a consequence of incarceration.

Also, we excluded women who did not hold a Norwegian PIN, which accounts for about one fourth of this population^45^. In general, this group largely comprise women from other countries with no residency in Norway and who are likely to either leave or be expelled from the country after their release from prison. To what extent they might be importantly different from the resident prison population with regards to mental health histories and other characteristics related to reoffending risk is unknown. Hence, there may be a systematic difference in who are included and excluded from this study and the results are only representative of the permanent resident Norwegian prison population.

## Conclusion

Women with severe mental illness or psychiatric comorbidities are overrepresented among those who reoffend after being released from prison. While this association can in part be explained by other measured and unmeasured characteristics, our analysis suggests that even after adjusting for a variety of other risk factors, women with a clinical history involving severe mental illness, multiple mental disorders involving substance use disorder or a history of multiple substance use disorders still presented with an elevated risk for reoffending.

## Data Availability

The ethical approval of this research project does not include permission to publicly share raw data. Qualifying researchers can apply for access to participant data with identifiers via the Norwegian Institute of Public Health and the Directorate of Norwegian Correctional Service upon approval from the regional committees for medical and health research ethics.
